# Cubic and hexagonal liquid crystal gels for ocular delivery with enhanced effect of pilocarpine nitrate on anti-glaucoma treatment

**DOI:** 10.1080/10717544.2019.1667451

**Published:** 2019-09-23

**Authors:** Wang Xingqi, Zhang Yong, Li Xing, Wang Yang, Huang Jie, Hu Rongfeng, Gui Shuangying, Chu Xiaoqin

**Affiliations:** a Department of Pharmaceutics, College of Pharmacy, Anhui University of Chinese Medicine, Hefei, People’s Republic of China;; b Institute of Pharmaceutics, Anhui Academy of Chinese Medicine, Hefei, People’s Republic of China

**Keywords:** Phytantriol, liquid crystal gels, ocular delivery, IOP-lowering, anti-glaucoma

## Abstract

The objective of this work was to investigate phytantriol-based liquid crystal (LC) gels including cubic (Q_2_) and hexagonal (H_2_) phase for ocular delivery of pilocarpine nitrate (PN) to treat glaucoma. The gels were produced by a vortex method and confirmed by crossed polarized light microscopy, small-angle X-ray scattering, and rheological measurements. Moreover, the release behaviors and permeation results of PN from the gels were estimated using *in vitro* studies. Finally, the anti-glaucoma effect of LC gels was evaluated by *in vivo* animal experiments. The inner structure of the gels was Pn3m-type Q_2_ and H_2_ phase, and both of them showed pseudoplastic fluid properties based on characterization techniques. *In vitro* release profiles suggested that PN could be sustainably released from LC gels within 48 h. Compared with eye drops, Q_2_ and H_2_ gel produces a 5.25-fold and 6.23-fold increase in the *P*_app_ value (*p* < .05), respectively, leading to a significant enhancement of corneal penetration. Furthermore, a good biocompatibility and longer residence time on precorneal for LC gels confirmed by *in vivo* animal experiment. Pharmacokinetic studies showed that LC gels could maintain PN concentration in aqueous humor for at least 12 h after administration and remarkably improve the bioavailability of drug. Additionally, *in vivo* pharmacodynamics studies indicated that LC gels had a more significant intraocular pressure-lowering and miotic effect compared to eye drops. These research findings hinted that LC gels would be a promising pharmaceutical strategy for ocular application to enhance the efficacy of anti-glaucoma.

## 1. Introduction

Glaucoma is a progressive optic neuropathy caused by retinal ganglion cell death. As a chronic ophthalmic disease, it is characterized by the elevation of the intraocular pressure (IOP), decrease of retinal sensitivity and optic nerve head changing that ultimately lead to decreased vision (Yu et al., 2015; Hu et al., 2018). High IOP is the leading risk factor for the development and progression of this disease, which could seriously lead to irreversible blindness. The elevation of IOP is caused by the imbalance between aqueous humor secretion and drainage (Zhao et al., 2017; Adams et al., 2018). Glaucoma is becoming a major cause of blindness and approximately 80 million individuals by 2020 have been affected worldwide (Hu et al., 2018).

Currently, lowering IOP by surgical procedures or pharmaceutical strategies to reduce eye damage is the chief treatment therapy for glaucoma (Zeng et al., 2018). In the field of pharmaceutics, ophthalmic delivery is facing many challenges due to its unique environment and physiological structure for pharmaceutical person (Tan et al., 2017). Conventional eye dosage forms, such as eye drops, have a low eye bioavailability ( < 5%) owing to tear washout, short retention time in the cul-de-sac and rapid tear turnover (Tian et al., 2018). To ameliorate the limitations of eye drops, various delivery approaches have been investigated to increase ocular bioavailability during the past decades, including two major strategies: to enhance the corneal permeability of the drugs and to extend the residence time of the formulations (Almeida et al., 2014; Yu et al., 2015). Building on such background, many investigators have attempted several novel drug delivery systems, for example, polymeric micelles (Zhou et al., 2017), inserts (Everaert et al., 2017), suspensions (Kontadakis et al., 2018), ointments (Patere et al., 2018), gels (Moustafa et al., 2018), and lipid-based nanocarriers (Liu et al., 2018), to improve eye bioavailability. Among above pharmaceutical techniques, various gel systems have attracted increasing attention for eye applications because they are more comfortable and likely to spread across the eye surface and tolerate the resist with tears during blinking (Krtalić et al., 2018).

Pilocarpine nitrate (PN), a miotic agent, has been applied to treat chronic glaucoma over 100 years. The pharmacological mechanism of PN is the ability to constrict the iris/ciliary body to regulate IOP, which further expands the trabecular meshwork to drain excess aqueous humor away from the anterior chamber (Chou et al., 2016). PN eye drop was mainly used as first-line anti-glaucoma drug in clinics, but their bioavailability is extremely low (less than 5% or even below 1%) due to the high hydrophilicity of PN (Nagarwal et al., 2009). Thus, it is required for frequent administration of a large amount of PN, approximately a drop each time and 3–6 times a day. This could cause various adverse effects, such as miosis and myopia, and may lead to systemic side effects, such as gastrointestinal irritation in more serious cases (Wang et al., 2017). In 2013, Khan et al. reported that PN-loaded nanosuspension as a drug carrier was applied to the eyes in order to improve the efficiency of PN at intraocular level and reduce the frequency of administration (Khan et al., 2013). Li et al. have researched in the same year liquid crystal (LC)-nanoparticles for ocular delivery of PN with the aim to enhancing the ocular bioavailability of drugs (Li et al., 2013). Most of these strategies provide some improvements over eye drops but the limitations of granular sensation and quick elimination are the major reasons that pharmaceutical workers have not widely accepted this (Wang et al., 2016; Krtalić et al., 2018).

Lyotropic LCs, and in particular reverse cubic (Q_2_) and reverse hexagonal (H_2_) phase, are good candidates as alternative delivery vehicles for a wide range of pharmaceutical applications. The unique inner morphology of Q_2_ and H_2_ phase enables embedment on a variety of drug molecules, such as hydrophilic, lipophilic and amphiphilic drugs (Mishraki et al., 2011). Lyotropic LCs form self-assembly by well biocompatible amphiphilic lipids, such as monoolein (MO), glyceryl monooleate (GMO), and phytantriol (PYT). PYT is one of the most widely studied amphiphiles creating the LCs’ matrices because of its nontoxic, well mucoadhesive, and biocompatible (Mishraki-Berkowitz et al., 2017; Yang et al., 2018). As the gel has good rheological characteristics, these lipid-based LCs were often selected for developing the gel-like preparations, which make them suitable for topical applications. Additionally, they are similar to the chemical and structural properties of living cell membranes with high mutual affinity. As such, these systems can be regarded as comfortable and promising drug delivery strategies (Yang et al., 2018). Recently, PYT-based LC gels were applied to the treatment of chronic diseases, such as periodontitis by injected into periodontal pocket (Cohen-Avrahami et al., 2010), rheumatoid arthritis for transdermal delivery (Wan et al., 2018) and liver cancer by injected into intra-arterial (Han et al., 2010), which have enhanced bioavailability and remarkably reduced systemic side effects.

Therefore, we developed biofilm-like lipid LC gels including Q_2_ and H_2_ phase for topical ocular delivery of PN to treat glaucoma in the present study. The gels were characterized by crossed polarized light microscopy (CPLM), small-angle X-ray scattering (SAXS) and rheological measurements. The *in vitro* drug release and *ex vivo* permeation experiments were used for *in vitro* evaluations. Furthermore, the biosafety and biocompatibility of the gels were studied by histological inspection. Pre-ocular retention time was performed by fluorescent labeling technology. Ultimately, *in vivo* pharmacokinetics and pharmacodynamics of PN in rabbits were investigated to prove that LC gels were able to extend drug residence time and strengthen therapeutic effect.

## 2. Materials and methods

### 2.1. Materials

PN (purity > 98.0%) was purchased from Shanghai TargetMol Biotechnology Co., Ltd. (Boston, MA). PYT (purity > 95.0%), triglyceride (TAG, purity > 99.0%), dexamethasone (purity > 98.0%), lidocaine (purity > 98.0%), fluorescein sodium, and l-glutathione oxidized (purity > 99.0%) were purchased from Shanghai Aladdin Biotechnology Co., Ltd. (Shanghai, China). Carbomer 940 (purity > 98.0%) was obtained from Shandong Yousuo Chemical Technology Co., Ltd (Jinan, China). Sodium pentobarbital (purity > 98.0%) was obtained from Shandong Xiya Chemical Industry Co., Ltd. (Shandong, China). PN eye drops was purchased from Shandong Doctor Lunfruida Pharmaceutical Co., Ltd. (Shandong, China). The purified water from the Milli-Q system (Millipore, Bedford, MA) was used in this study. All other chemicals and reagents were of analytical grade or higher in the whole experiment.

Male New Zealand albino rabbits (2.0–3.0 kg) were supplied by Animal Experimental Center of Anhui University of Chinese Medicine (Hefei, China). All animal experiments were performed in accordance with the guidelines approved by the ethics committee of Anhui University of Chinese medicine (Hefei, China).

### 2.2. Preparation of LC gels

#### 2.2.1. Preparation of PN-loaded Q_2_ gel

The Q_2_ gel (FQ) used in the studies comprised 70 wt% PYT and 30 wt% water. First, appropriate quantities of PYT was heated at 60 ± 0.5 °C as an oily phase. Then, PN (3 wt%, with respect to PYT/water binary system) was placed in the water at the same temperature to prepare an aqueous phase. We calculated the maximum drug loading was 3 wt% according to the solubility of PN (146.87 mg/mL) to maximize the content of drug in the formulation. Finally, the aqueous phase at 60 ± 0.5 °C was moved to the oily phase while vortex-mixed for 5 min. The gels were retained in a small centrifugal tube and equilibrated for one week at room temperature.

#### 2.2.2. Preparation of PN-loaded H_2_ gel

TAG containing medium-chain fatty acids can solvate the lipid tails, affecting the critical packing parameters of LCs and converting the lamellar (Lα) and Q_2_ phases into an H_2_ phase that can be formed and maintain stability at ambient temperature (Libster et al., 2007). Formulations of the H_2_ gel (FH) contained PYT, TAG and water (71.15:3.85:25, w/w/w). The preparation method was the same as that used for FQ except dissolving TAG into the PYT as an oily phase to induce H_2_ phase.

### 2.3. Characterizations of the formulations

#### 2.3.1. CPLM and SAXS observation

The internal morphology of the LC gels was observed by placing a few samples on glass slides and viewing them under CPLM (CK-500, Shanghai Caikon Optical Instrument Co., Ltd., Shanghai, China). Then, images of the formulations were taken by computer.

Further structure analysis and phase identification of LC gels were carried out by SAXS measurement (Anton Paar, Graz, Austria) at room temperature. The samples were tested at 40 kV and 50 mA for 10 min using an X-ray source (Cu Kα radiation, *k* = 0.154 nm). Scattering message was recorded on the image board with aluminum foil as background, and then the scattering file subtracts the scattering information of aluminum. The scattering factor (*q*) of the Bragg peaks at 0.1–5.0 nm^–1^ and the scattering intensities were plotted versus the *q*. The specific parameters of LCs were computed by the following equations (Mei et al., 2017):

d=2π/q

$$a=(h^{2}+k^{2}+l^{2})1/2d$$

$$R_{w}=a{\left[(\sqrt{3}/2\pi )(1-\Phi_{s})\right]}^{\frac{1}{2}}$$

where d means the distance of reflection space between LCs planes, a represents the lattice parameter of LCs which hints the size of aqueous channels in the inner structure, h, k, and l are Ptolemy indexes and have no dimensions, $R_{w}$ is the radius of water channels, and $\Phi_{s}$ represents the volume fraction of surfactant.

#### 2.3.2. Rheological study

A DHR-2 rheometer (TA Instruments, New Castle, DE) was used to evaluate the rheological characteristics of LC gels and incorporate a cone-plate sensor with a cone angle of 1° and a diameter of 20 mm. The 200 mg sample was loaded onto the sample table and the sensor was subsequently adjusted to acquire the required measurement gap. The sample was balanced at the measured temperature for two minutes before starting the measurement procedure.

Flow scanning mode was carried out to evaluate the viscosity of PN-loaded LC gels in the flow test, and the shear rate procedure was in the range of 0.1–100 s^–1^, while the temperature was maintained at 37 ± 0.1 °C.

The linear viscoelastic domain of the gels was determined by oscillating stress scanning at a fixed frequency (1 Hz) before performing the oscillation measurements. The amplitude scanning measurements were performed at a strain range from 0.01 to 15%. In the range of 0.01–100 rad·s^–1^, frequency sweep tests were carried out using the oscillation-frequency mode at 37 ± 0.1 °C based on a value of the linear viscoelastic domain. The viscoelasticity of the gels was expressed by the elastic modulus (*G*′) and the viscous modulus (*G*″).

### 2.4. *In vitro* drug release

The dialysis membrane (10–12 kDa, Shanghai, China) was pre-immersed for 30 min and then washed thoroughly with deionized water to evaluate the drug release behavior. Subsequently, 50 mg of each of the LC gels containing 1.50 mg PN was placed in the dialysis membrane and kept in the oven to keep the eye surface temperature of 35 °C for 30 min. Meanwhile, 150 μL of eye drops containing 1.50 mg PN was used as the control group. Then, the resulting membrane was immersed in 12 mL simulated tear (STF, pH = 7.2, 6.78 g NaCl, 2.18 g NaHCO_3_, 0.084 g CaCl_2_·2H_2_O, and 1.38 g KCl in 1000 mL of purified water) at 35 ± 0.5 °C with stirring at 100 rpm. At predetermined times, including 0.33, 0.67, 1.00, 1.50, 2.00, 2.50, 3.50, 4.50, 5.50, 7.50, 9.50, 11.50, 24, 36, and 48 h, 1 mL of the release sample was taken out from the dissolution flasks and the same amount of fresh medium was added back. The collected samples were determined using HPLC technique. The cumulative amount of drug (*Q_n_*, μg) was computed using the following equation:

$$Q_{n}=c_{n}\times v_{0}+\sum_{i=1}^{n-1}c_{i}\times v_{i}$$

In the equation, $c_{n}$ means the PN concentration of the dissolution medium at different sampling points; $v_{0}$ and $v_{i}$ are the volumes of the release medium and the sample, respectively; $c_{i}$ represents the drug concentration of the *i*th sample.

The drug release kinetics and mechanism of different models were fitted, and the best fitting kinetics model with the highest correlation coefficient was obtained.

### 2.5. *Ex vivo* corneal penetration studies

The freshly excised corneas were obtained from New Zealand albino rabbit’ eyes for the *ex vivo* experiments. The excised cornea should be immediately conserved in a glutathione bicarbonate ringer (GBR) buffer at 35 ± 0.5 °C. The cornea with an effective diffusion area of 0.71 cm^2^ was mounted on the modified Franz cells, which consisted of a receptor and a donor compartment with the epithelial side facing the donor chamber. Fifty milligrams of LC gels (containing 1.50 mg PN) and 10.4 mL of GBR buffer were placed into the donor and receptor compartment, respectively. Simultaneously, 150 μL PN eye drops (containing 1.50 mg PN) was used as the control group. At specified times (30, 60, 90, 150, 210, 270, 330, 390, and 450 min), 1 mL release medium was collected from the receptor compartment and substituted for an equivalent volume of fresh medium to keep the initial volume. Each experiment was conducted three times. The PN content that permeated across rabbit corneas was analyzed by HPLC technique.

The apparent permeability coefficient (*P*_app_, cm/s) was calculated using the following equations (Vázquez-González et al., 2014):

$$P_{\text{app}}=\frac{\Delta Q}{\Delta t\times C_{0}\times A\times 60}$$

$$J_{\text{ss}}=C_{0}\times P_{\text{app}}$$

where ΔQ/Δt stands for the steady-state of the linear portion of the plot of the amount of PN (Q/μg) in the receptor chamber vs. time, $C_{0}$ means the original PN loading concentration (μg/mL) in the donor compartment, A represents the corneal effective surface area (0.71 cm^2^), and 60 is the conversion of units from minutes to seconds (s).

### 2.6. Corneal hydration levels

In order to estimate the irritation of the formulations to cornea, the corneal hydration level (HL) was investigated after the corneal permeation experiment. Each corneal sample was gently removed from the scleral ring and weighed (*W*_w_). Whereafter, the samples were reweighed (*W*_d_) after desiccation at 70 ± 0.5 °C for 12 h. The HL% value was calculated using the following equation (Yu et al., 2015):

$$\text{HL}\%=\left[1-\left(\frac{W_{d}}{W_{w}}\right)\right]\times 100$$

### 2.7. *In vivo* animal experiments

#### 2.7.1. Pre-ocular resident time evaluation

In this experiment, sodium fluorescein-loaded formulations (i.e. LC gels and solution) were prepared to evaluate their *in vivo* preocular residence according to a previously reported protocol (Wang et al., 2016). Hydrophilic PN was replaced by 1 wt% sodium fluorescein in the aqueous phase and then processed using the same method as for the LC gels preparation. Meanwhile, 1 wt% sodium fluorescein-loaded solution was used as a reference. Then, 50 mg LC gels and 50 μL solution (all contain 0.50 mg of sodium fluorescein) were applied to the lower fornix of rabbit eyes. Then, the intensity of fluorescein was monitored at a predetermined time point using blue-light-activated fluorescent lamp.

#### 2.7.2. Ocular histology

In order to inspect the effect of the formulations on ocular tissues structure and integrity, all rabbits were killed through the ear vein injection of air after a week of long-term administration (three times a day). Normal saline was used in the control group. Ocular tissue samples were instantly exenterated, washed with normal saline, and soaked in neutral buffered formalin solution 10% (v/v) for 24 h. Then, the paraffin-embedded tissues were cut into 5 um sections and stained with hematoxylin and eosin (H&E). These histological sections, including cornea, iris, and retina, were observed using an optical microscope (BM-37XB, Shanghai Piam Optical Instrument Manufacturing Co., Ltd, Shanghai, China) and taken the histologic photographs for toxicity inspection.

#### 2.7.3. Pharmacokinetic studies in aqueous humor

Nine healthy New Zealand white rabbits (2.0–3.0 kg) were used as animal model to estimate the pharmacokinetics of PN in aqueous humor. Rabbits were randomly divided into three groups with three rabbits in each group. Each formulation, 50 mg FH, 50 mg FQ, and 150 μL eye drops (all contain 1.50 mg of PN), was treated with the lower conjunctival sac of the rabbit’s eye (two eyes), and the eyelids were gently held closed for 10 s to maximize contact between the drug and the cornea. After 0.17, 0.33, 0.50, 0.75, 1, 2, 4, 6, 8, 10, and 12 h of administration, samples of 100 μL aqueous humor were gathered from the eyes using the 1 mL injection syringe. Aqueous humor samples were mixed with 100 μL HPLC mobile phase to dislodge the protein by vortexed for 1 min. These samples were then centrifuged at 4000 rpm/min for 10 min to obtain supernatants. Finally, PN concentration in aqueous humor was analyzed using HPLC technique. All pharmacokinetic data analysis was carried out using DAS 2.0 (Shanghai Bojia Pharmaceutical Technology Co., Ltd., Shanghai, China).

#### 2.7.4. Pharmacodynamics studies in normal rabbits

##### 2.7.4.1. Pupillary effect

The miosis tests were carried out using 12 New Zealand albino rabbits (2.0–3.0 kg) and randomly divided into four groups with three rabbits in each group. All experiments were conducted after adapting to the environment in a room under standard lighting conditions. The pupil diameter was gauged using a pupil ruler with an increment of 0.5 mm under standardized conditions. The basal measurements of the pupil diameter were recorded as $d_{0}$ at zero time (before application) and measured four readings in each eye. The tested formulations (50 mg LC gels, 150 μL eye drops, all contain 1.50 mg of PN) were then administered into the lower fornix of rabbit’s right eye, while left eye was dripped into normal saline as a control. At a specified time intervals, the pupil diameters were measured as $d_{t}$ after application. Miotic response (*M*) can be computed as follows: $M=(d_{0}-d_{t})/d_{0}\times 100\%,$ and the miotic response versus time profiles was plotted.

##### 2.7.4.2. IOP Measurements

The PN-loaded gels were investigated for their reduction IOP effects on New Zealand white rabbits and the outcomes were compared with that of PN eye drops as well as normal saline. The rabbit IOP was gauged by an indentation tonometer (YZ7, Suzhou Medical Apparatus Factory, Suzhou, China). Before measurement, the rabbit eyes were locally anesthetized with 35 μL of a 0.2% (w/v) lidocaine hydrochloride. First, the baseline IOP of each rabbit’s eyes was gauged before the application of formulations. Then, the lower conjunctival sac of right eye of each rabbit (*n* = 3) was administered with 50 mg LC gels, 150 μL eye drops (all contain 1.50 mg of PN) and the eye was manually closed for 30 s, while the left eye received 50 μL normal saline as a reference. After administration, at predetermined times, the rabbits were placed in restricted bags with its head exposed and the IOP was measured. The percentage decline in IOP was calculated by the following equation:

$$\%\;\text{Decrease}\;\text{in}\;\text{IOP}=\left({\text{IOP}}_{\text{initial}}-{\;\text{IOP}}_{\text{scheduled}\;\text{time}}\right)/{\text{IOP}}_{\text{initial}}\times \;100\%$$

All data are recorded with the same tonometer, at least three eyes were measured, and the mean values at each time for each formulation were calculated.

#### 2.7.5. Pharmacodynamics studies in glaucomatous model

Twelve adult New Zealand white rabbits (2.0–3.0 kg) were used for the establishment of glaucoma model. Before the experiment, a slit lamp was used to inspect the eye surface to ensure ocular health. Before modeling, the rabbit eyes received 35 μL Alcaine^®^ eyedrops for local anesthesia, and normal IOP measurements were performed by using an indentation tonometer and five readings were averaged. Both 0.025% (w/v) dexamethasone and 0.3% (w/v) carbomer 940 were dissolved in normal saline as an inducer of high IOP. Then, 100 μL blank aqueous humor was collected from the anterior chamber using a 1 mL injection syringe and substituted for 100 μL inducer into the eyes. In order to effectively prevent infection, the rabbit eyes were administrated with TobraDex^®^ eye drops every day during the establishment of the model. IOP was monitored daily for one week after induction until IOP exceeded normal IOP value (21 mm Hg) for three consecutive days, indicating the successful construction of the model. The therapeutic efficacy of anti-glaucoma, including miosis tests and IOP measurements, was carried out as described previously for two weeks.

### 2.8. Statistical analysis

All experimental data were reported as mean ± SD and analyzed with the software program Origin Pro 8.5. Comparisons between the different groups were determined by Student’s *t*-test using IBM SPSS Statistic 23 software (IBM SPSS Statistics, Armonk, NY). *p* < .05 was taken as the statistical significant difference.

## 3. Results and discussion

**Figure 1.**
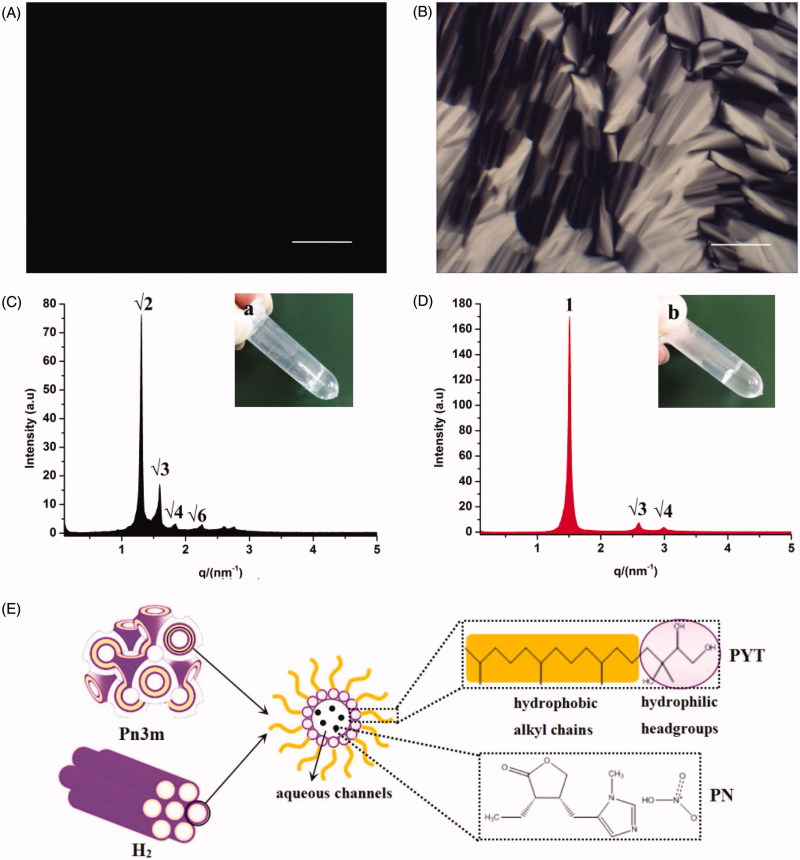
Images of FQ (A) and FH (B) formulations under CPLM (all images were taken at ×100 magnification and the scale bar is 100 μm). SAXS profiles of FQ (C) and FH (D) formulations at 25 ± 0.5 °C, appearance pictures of FQ (a) and FH (b) formulations at room temperature. (E) Schematic illustration of the inner structure of Pn3m-type Q_2_ and H_2_ phases containing PN molecules.

According to the relevant literature, we chose Q_2_ and H_2_ phase LCs because they have a better sustained release effect. In 2003, Barauskas and Landh reported the phase diagram of PYT/water binary system (Barauskas and Landh, 2003). Later, the phase diagram of glycerol monooleate/TAG/water ternary system was reported (Libster et al., 2007). In addition, we fully considered the solubility of PN and the basis of our previous work to select the ratio of formulations (Chu et al., 2018). Hence, PYT/water (70:30, w/w) binary system and PYT/TAG/water (71.15:3.85:25, w/w/w) ternary system were selected to prepare 3 wt% PN-loaded Q_2_ and H_2_ gels, respectively. As displayed in Figure 1(a,b), it was clearly monitored that drug-loaded LC gels were clarified and transparent gel-like formulations at ambient temperature suitable for ocular administrations.

### 3.1. CPLM and SAXS analysis

CPLM was used to evaluate the micrograph morphology of the formulations as exhibited in Figure 1. The phase structure of LCs can be distinguished by the optical texture in CPLM. As shown in Figure 1(A), a dark view without birefringence hints that FQ may be a Q_2_ phase (Chu et al., 2018). Nevertheless, a clear fan-like, angular texture in Figure 1(B) was the morphology mostly observed in FH, indicating that the formulation has formed an H_2_ phase (Ki et al., 2014).

In order to further confirm the LC nanostructures of the gels containing PN, SAXS technique was carried out in this paper. The nanostructures of the LCs could be identified according to the ratio of the *q* corresponding to scattering peaks in the SAXS curves. The representative SAXS pattern was displayed in Figure 1, with respective relative ratios of FQ and FH as $\sqrt{2}:$$\sqrt{3}:$$\sqrt{4}$… (Figure 1(C)) and 1:$\sqrt{3}:$$\sqrt{4}$… (Figure 1(D)), respectively. Therefore, a double diamond Q_2_ (Pn3m) and H_2_ phase was deemed to be the LC nanostructure of the FQ and FH, respectively. These outcomes were in good agreement with the CPLM observations (Figure 1(A,B)).

The Pn3m inner structure consists of a bi-continuous aqueous channel and a curved lipid bilayer extending in three-dimensional and dense network nanostructure. The high viscosity and retarded drug release of Q_2_ phase may be mainly attributed to these complex LC nanostructures. The H_2_ phase was composed of several rod-like micelle cylinders lying parallel to each other and comparatively enclosed, which are hexagonal arrays as depicted in Figure 1(E) (Mei et al., 2017). It was reported that the main fraction of hydrophilic PN molecules were chiefly distributed in the water cores or combined with polar headgroups of the PYT (Rahanyan-Kägi et al., 2015). As listed in Table 1, the calculated a value of the nanostructures of FQ and FH was 59.69 and 48.73 Å, respectively. Obviously, Q_2_ gel has a larger values (*p* < .01) than H_2_ gel. Meanwhile, the $R_{w}$ value of Q_2_ phase was significantly (*p* < .05) higher than that of H_2_ phase. It can be therefore inferred that the release rate of hydrophilic molecules in Q_2_ phase was faster than that in H_2_ phase, and the sustained release effect of H_2_ phase was comparatively better for hydrophilic drugs.

**Table 1.** The critical parameters of SAXS results were calculated using the relevant formulas.

Formulations	Space group	Bragg peaks	a (Å^a^)	d (Å)	$R_{w}$ (Å)
FQ	Pn3m	√2:√3:√4…	59.69 ± 0.70**	42.20 ± 0.73	83.76 ± 0.78*
FH	H_2_	1:√3:√4…	48.73 ± 0.61	41.42 ± 0.84	79.16 ± 0.49

a 1 Å=10^–10^ m.

*p<.05, statistically significant compared with FH.

**p<.01, statistically significant difference between FQ and FH.

### 3.2. Rheological properties

**Figure 2.**
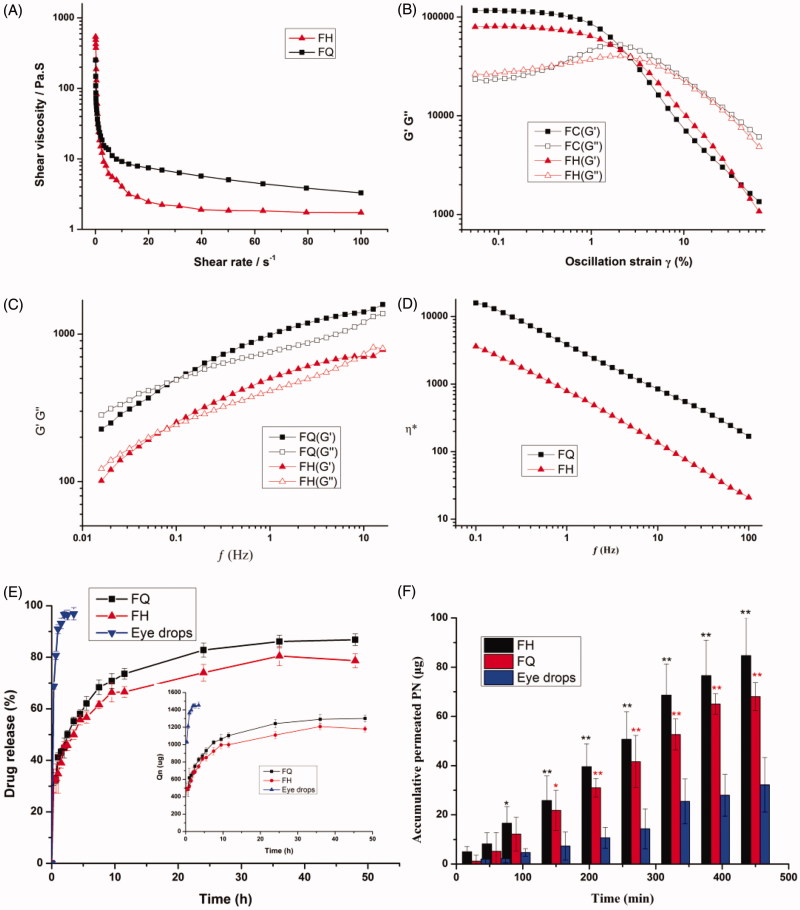
(A) Flow curves showing the effect of shear rate sweep on viscosity of the LC gels. (B) Rheological profiles of LC gels were evaluated by strain sweep at 0.01–100%. (C) Viscous and elastic moduli dependence upon oscillation frequency for LC gels. (D) The complex viscosity of LC gels at 37 ± 0.5 °C as a function of angular frequency. (E) The cumulative release profiles of PN from different formulations. The inset panel exhibits the drug release flux graph. (F) *Ex vivo* transcorneal permeation profiles of PN from LC gels and eye drops using fresh rabbit corneas. Data are reported as mean ± SD of *n* = 3. *p<.05, statistically significant compared with eye drops. **p<.01, compared with eye drops.

Viscosity is one of the pivotal attributes for novel ocular formulations defining drug release and the retention time at the eye surface. As shown in Figure 2(A), the viscosity vs. shear rate was plotted to evaluate the flow characteristics of the gels. The viscosity profile of LC gels tested at the shear rate of 1–100 s^–1^ decreases with the increase of shear rate, implying that the formulations showed shear-thinning nature like pseudoplastic fluid. Due to the low viscosity during blinking and the high viscosity during interblinking, shear thinning behavior was in favor of uniform spreadability after application to the eye surface (Patere et al., 2018). Furthermore, this nature could be promising for ophthalmic administration to shield the encapsulated corneal cells from shear forces (Mokhtari et al., 2019).

The linear viscoelastic shear strain gradient ranges from 0.01% to 2.0%, and the optimum strain value was 2.0% as exhibited in Figure 2(B). This provides information about the dynamic characteristics of elasticity modulus *G*′ and viscous modulus *G*″. The inherent properties of LC gels at physiological temperatures can be provided by the dynamic characteristics of the gels, such as *G*′ and viscosity *G*″. The outcomes of the rheological analyses reveal the viscoelastic properties of the gels at low frequencies ( < 0.1 Hz), where *G*′ < *G*″ and a frequency dependence was monitored (Figure 2(C)). Under this situation, the viscosity modulus is predominant, hinting that the less irritation the gels would impose to the surrounding ocular tissues. However, when the frequency is higher than 0.5 Hz, the gels displayed dominant solid-like nature (*G*′>*G*″) to resist damage of the shear force of blinking period to retain the structure integrality of depot carrier and prolong residence time (Mei et al., 2017).

Application and *in vivo* performance of the formulations were greatly affected by the viscosity under different conditions. As illustrated in Figure 2(D), the complex viscosity (η*) values in the LC gels decreases with applied angular frequency, which proves the shear-thinning property. The H_2_ gels exhibited a lower η* value, with characteristics of pseudoplastic fluids, indicating that H_2_ gels could be more evenly distributed on the ocular surface. The η* value of Q_2_ gel had a significantly higher viscosity (*p* < .05) compared to H_2_ gel at an identical angular frequency, implying the internal architecture of Q_2_ gel may be more stable, which is conducive to the storage and release of drugs.

### 3.3. *In vitro* release profile and release kinetics

Figure 2(E) displays the percentage of PN entrapped in the LC gels determined using HPLC technique. Obviously, the amount of PN released from LC gels at the first point was 32.77 ± 2.43% and 32.09 ± 4.56% for FQ and FH, respectively, in contrast to 69.59 ± 1.00% for eye drops. Compared with the corresponding LC gels, the drug release increased more than 2-folds from the eye drops. PN in LC gels showed significant (*p* < .05) retardation in the initial drug release compared to conventional eye drops. It could be clearly monitored that PN was almost entirely released (96.87 ± 2.41%) from eye drops within 3.5 h, which indicates eye drops has a burst release effect. The high drug release observed from the eye drops may be due to the low liquid consistency resulting in rapid drug leakage. Nevertheless, the LC gels exhibited a prolonged release ranging from 78.66 ± 2.71 to 86.80 ± 2.28% within 48 h, revealing that PN molecules were strongly enveloped in the water cylinders of LC gels. By comparing the percentage of PN released from FQ and FH containing identical PN, it was found that the cumulative release of FQ was significantly greater (*p* < .05) than that of FH after 6 h. These results were in good agreement with the SAXS outcomes, hinting the a and $R_{w}$ values of LCs have a remarkably effect on drug release. It was inferred that the larger the value of a and $R_{w},$ the more drug release, on the contrary, the less drug release. These release data attested that Q_2_ and H_2_ gels could maintain drug release by their unique internal structure leading to a decrease in the dosing frequency to improve patient compliance.

Various mathematical models were fitted to understand the mechanism of PN release in STF; the results are listed in Table 2. The drug release behaviors of all tested LC gels were best fitted with the Higuchi model (*R*^2^=0.9762–0.9869), hinting a diffusion controlled release. The LC gels displayed *n* values  <  0.45, indicating that they followed the Fickian diffusion mechanism according to the Ritger and Peppas (Fan et al., 2016).

**Table 2.** Kinetics of releasing LC gels by fitting different mechanism models.

	Zero order	First order	Higuchi model	Ritger–Peppas	
Formulations	*R*^2^	*R*^2^	*R*^2^	*R*^2^	*n*
FQ	0.8621 ± 0.038	0.8324 ± 0.021	0.9345 ± 0.027	0.9762 ± 0.035	0.3112 ± 0.022
FH	0.7658 ± 0.017	0.8774 ± 0.036	0.8832 ± 0.021	0.9869 ± 0.028	0.2541 ± 0.013

All results are represented as mean ± SD of *n* = 6.

### 3.4. *Ex vivo* corneal permeation and hydration evaluations

Figure 2(F) presents the transcorneal permeation profiles of LC gels and PN eye drops *ex vivo*. Obviously, a comparatively high permeability was obtained in the LC gels group in comparison to eye drops. Meanwhile, the transcorneal penetration parameters are summarized in Table 3. It can be observed that the *P*_app_ value of Q_2_ and H_2_ gels was 5.25-fold and 6.23-fold more than PN eye drops, respectively, which led to a significant elevation (*p* < .05) of PN absorption through the cornea. This may be due to the good biocompatibility between biofilm-like lipid carriers and corneal epithelial cells, resulting in more easily transport and an elevated solubility of encapsulated drug molecules through the corneal barriers. Besides, PYT, as an amphiphilic lipid, has its own osmotic promoting effect could penetrate across tight junctions to strengthen corneal epithelial permeability (Zhang et al., 2015; Chu et al., 2018; Wan et al., 2018). Surprisingly, the corneal permeability of H_2_ gel is better than that of Q_2_ gel, which is contrary to the results of *in vitro* release. This might be due to the fact that rigidity and viscosity of H_2_ gel are lower than that of Q_2_ gel, leading to an efficient transport of hydrophilic PN through the cornea. These outcomes were coincident with our rheological analysis. However, we need to further carry out *in vivo* animal experiments in order to evaluate the anti-glaucoma effect of LC gel.

**Table 3.** Transcorneal permeation parameters of LC gels and eye drops *ex vivo*.

Formulations	*J*_ss_×10^2^/(μg·s^–1^ cm^-2^)	*P*_app_×10^5^/(cm·s^–1^)	HL%
FQ	47.50 ± 0.06*	4.99 ± 0.61*	78.64 ± 0.38
FH	56.36 ± 0.06**	5.92 ± 0.67**	77.86 ± 0.25
Eye drops	9.06 ± 0.02	0.95 ± 0.07	76.71 ± 0.52

All data are reported as mean values ± SD, *n* = 3.

*p<.05, difference from eye drops.

**p<.01, statistically significant compared with eye drops.

Corneal HLs were used to estimate the degree of any corneal injury *in vitro*. The HLs of healthy cornea were 76–80%, and when an HL value higher than 83% was associated with a certain degree of injury to the epithelium and/or endothelium (Moustafa et al., 2018). The HL value of three formulations is summarized in Table 3. The outcomes revealed that LC gels produced no significant corneal irritation for ocular application.

### 3.5. *In vivo* preocular retention

**Figure 3.**
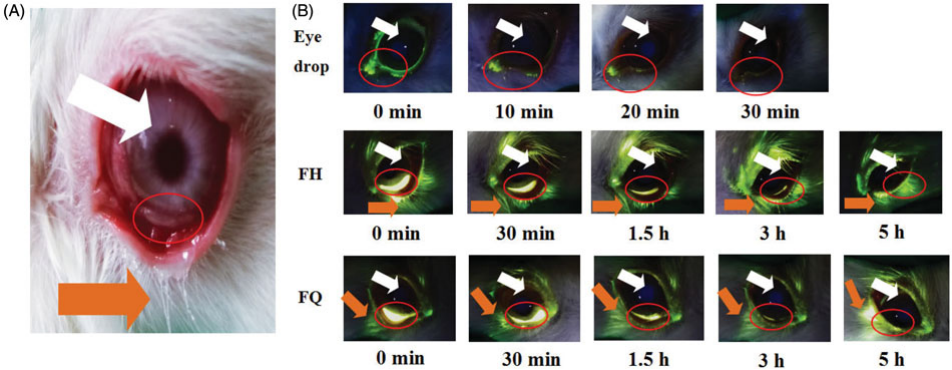
(A) The lower fornix of the rabbits’ eyes and the anterior surface of the eyeball. (B) Fluorescence photographs of rabbit eyes after application of sodium fluorescein-loaded formulations. Orange and white arrows indicate the release of sodium fluorescein by the gels and the location of the eyeball, respectively.

In order to estimate the intraocular residence time *in vivo*, sodium fluorescein-loaded H_2_ gel and Q_2_ gel were applied to the lower fornix of the rabbit eye, and photos were recorded at a predetermined time point (Figure 3). In this experiment, sodium fluorescein-loaded solution was used to represent eye drops as a reference. It could be found that the quite strong signal intensity was observed for LC gels, and most of this signal was distributed in the lower conjunctival sac even more than 3 h, i.e. the site of administration, compared with eye drops. Obviously, the rabbit eye which received eye drops did not show a strong signal intensity and rapidly cleared after 10 min of administration, indicating that eye drops had a short retention time on the ocular surface. It was indicated that LC gels had well bioadhesion property to overcome blinking and flushing of tears, which may be due to the interaction between biofilm-like LC gels and mucin in corneal epithelium. These results were in line with our rheological analysis.

### 3.6. Histological inspection

**Figure 4.**
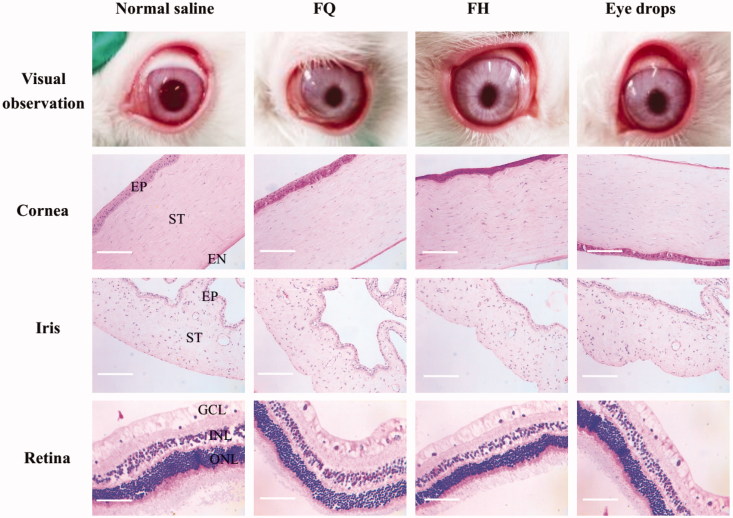
Representative histological images of the eye tissues after treated with various formulations for a week (original magnification, ×200). EP: epithelium; ST: stroma; EN: endothelium; ONL: outer nuclear layer; INL: inner nuclear layer; GCL: ganglion cell layer.

Ocular tissue cross-sections, including cornea, iris, and retina, were inspected after seven days of application to evaluate the influence of LC gels on the tissue integrity and corneal cell structure. As exhibited in Figure 4, a typical stratified epithelial layer can be identified by protruding at the nuclei of the squamous surface cells and the basal columnar cells. The epithelium and stroma structure of corneas treated with LC gels and eye drops were almost no change compared with the normal saline group. These corneas have defined and smooth structures, and no obvious irritation symptoms were examined in the iris and retina. It was therefore proved that LC gels have good biocompatibility and can be administrated with the eye for a long-term to treat chronic diseases.

### 3.7. Pharmacokinetics study

**Figure 5.**
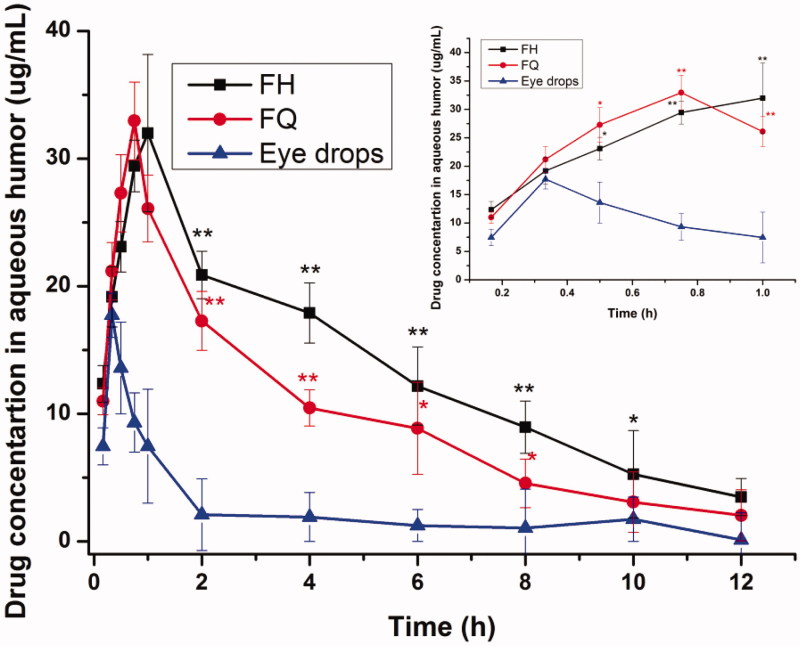
PN concentration in rabbits’ aqueous humor at different time points after administration of LC gels and a commercial PN eye drops (all outcomes are reported as mean ± SD of *n* = 3). *p<.05, statistically significant compared with eye drops. **p<.01, compared with eye drops.

In order to monitor the PN release properties in local eye site, *in vivo* pharmacokinetics in aqueous humor was investigated in this section. Figure 5 displays the concentration of PN in normal rabbit’s aqueous humor after application of various formulations. It was observed that after 12 h, the PN in aqueous humor could still be examined in LC gel group, while unexaminable in the eye drops group. Moreover, the concentration of PN in gel group was relatively stable, and the concentration in the later stage was prolonged. As listed in Table 4, the main pharmacokinetic parameters of PN in aqueous humor were summarized using DAS 2.0. The maximum PN concentration (*C*_max_) of PN in rabbit’s aqueous humor was achieved at 0.75 h and 1.00 h after application of FQ (*C*_max_=32.95 ± 5.74 mg/L) and FH (*C*_max_=31.99 ± 4.60 mg/L), respectively, which was about 1.86-fold and 1.80-fold higher than that of administration of PN eye drops at 0.33 h (*C*_max_=17.73 ± 5.40 mg/L). Compared with the traditional eye drops, the AUC_0–12 h_ value was enhanced by 6.03-fold and 4.32-fold for FH and FQ, respectively. Meanwhile, the MRT value of LC gels was longer than that of eye drops. There were significant differences (*p* < .05) in the *C*_max_, AUC_0–12 h_ and MRT values of LC gels compared with the corresponding values for the eye drops.

**Table 4.** The key pharmacokinetic parameters of PN in aqueous humor after local application in the normal rabbits ($\bar{x}$±SD; *n* = 3).

Key parameters	FQ	FH	Eye drops
AUC_0–12 h_ (mg/L/h)	130.62 ± 17.45**	182.31 ± 23.15**	30.22 ± 6.41
*C*_max_ (mg/L)	32.95 ± 5.74*	31.99 ± 4.60*	17.73 ± 5.40
*T*_max_ (h)	0.75	1.00	0.33
MRT (h)	4.63 ± 1.32*	5.14 ± 1.03*	3.79 ± 0.92

*p<.05, statistically significant compared with eye drops.

**p<.01, compared with eye drops.

The higher ocular bioavailability provided by the LC delivery system may be due to three factors, namely, drug permeability through the cornea, a prolonged ocular contact time on the corneal epithelial layer and a sustained-release effect. These results suggested that the hydrophilic PN molecules would maximize its release from the aqueous channels of biofilm-like LCs through the corneal epithelial membrane to enhance therapeutic efficacy. Therefore, biofilm-like LC gels as novel ophthalmic formulation could reduce medication frequency and associated side effects and improve patient compliance.

### 3.8. Pharmacodynamics of normal rabbits

#### 3.8.1. Miotic effect

**Figure 6.**
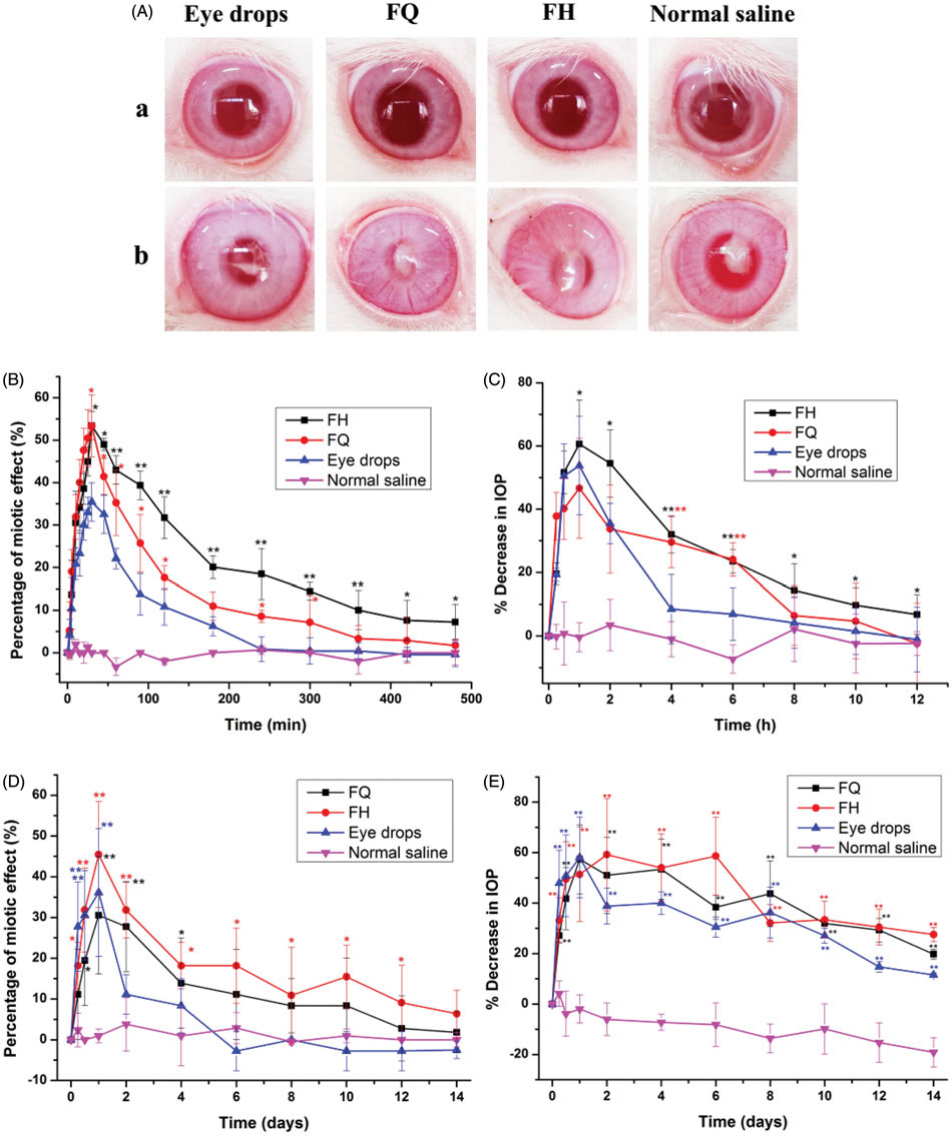
(A) Ocular surface was inspected by a slit lamp (a) before establishment of model and (b) after treatment with various formulations. Time-course measurements of (B) pupil diameter and (C) IOP after administration of different PN-loaded formulations in normal rabbits. Time-course measurements of (D) IOP and (E) pupil diameter after administration of different preparations for two weeks in glaucomatous rabbits. *p<.05, statistically significant difference from eye drops. **p<.01, statistically significant compared with eye drops.

The pupil response curves of PN released from different formulations within 8 h are exhibited in Figure 6(B). Meanwhile, the key pharmacodynamic parameters of miosis were calculated by logarithmic trapezoidal method as summarized in Table 4. It was found that the AUC_0–480 min_ values after administration for H_2_ gel and Q_2_ gel were increased by 3.06-folds and 1.96-folds, respectively, compared to the eye drops, indicating that the eye bioavailability of LC gels were significantly (*p* < .05) than that of traditional eye drops. The maximum miosis rates of H_2_ gel and Q_2_ gel were 53.41 ± 3.42% and 53.33 ± 6.05%, respectively, after application, and LC gels have longer MRT (*p* < .05) for miosis effect than eye drops. These results suggested that LC gels have more notable extended effect on miosis compared with eye drops. This was in line with the outcomes for the *ex vivo* corneal penetration as well as precorneal residence time studies, where PYT-based biofilm-like gels exhibited the good performance for ocular application (Table 5).

**Table 5.** The key pharmacodynamic parameters of miotic response of various formulations were summarized (the results represent mean values ± SD, *n* = 3).

	Pharmacodynamic parameters			
Formulations	Peak (%)	*T*_max_ (min)	AUC_0–480 min_	MRT (min)
FH	53.41 ± 3.42*	30.00	10102.06 ± 2076.14**	163.82 ± 33.59**
FQ	53.33 ± 6.05*	30.00	6465.13 ± 1632.49*	125.46 ± 51.25*
Eye drops	35.42 ± 3.39	30.00	3296.09 ± 786.23	80.14 ± 15.60

*p<.05, statistically significant compared with eye drops.

**p<.01, compared with eye drops.

#### 3.8.2. Reduction IOP effect

Figure 6(C) depicts the change in the percentage of IOP measured in normal rabbits as a function of time after application with various formulations. Furthermore, some main pharmacodynamic parameters were listed in Table 6. Generally, an average decrease of 15% or higher was considered to be valid in IOP control (Boyd et al., 2006). Obviously, normal saline was devoid of remarkable change in IOP as a control group. However, the mean maximum percentage reduction in IOP by 46.64 ± 11.79%, 60.61 ± 17.04%, and 53.81 ± 14.96% occurred in 1 h after administration of FQ, FH, and eye drops, respectively, revealing H_2_ gel showed a stronger effect on IOP lowering in comparison with others. The AUC_0–12 h_ values of H_2_ gel and Q_2_ gel were 1264.34 ± 180.87 and 868.67 ± 148.07, respectively, which hinted that the ocular efficiency of LC gels was greater (*p* < .05) than that of commercial product (159.45 ± 39.50). Taking into account the duration of IOP lowering, the effect of LC gels obviously continued over 12 h, while the market product lasted for only 4 h. As listed in Table 6, LC gels have higher MRT values (*p* < .05) for percentage reduction in IOP than eye drops.

**Table 6.** The main pharmacodynamic parameters after application of LC gels and traditional eye drops (all results are represented as mean ± SD, *n* = 3).

	Pharmacodynamic parameters			
Formulations	Max % decrease in IOP	*T*_max_ (h)	AUC_0–12 h_	MRT (h)
FQ	46.64 ± 11.79	1.00	868.67 ± 148.07**	3.62 ± 0.87
FH	60.61 ± 17.04	1.00	1264.34 ± 180.87**	4.00 ± 1.65*
Eye drops	53.81 ± 14.96	1.00	159.45 ± 39.50	2.54 ± 0.31

*p<.05, statistically significant compared with eye drops.

**p<.01, compared with eye drops.

It could be therefore concluded that the IOP reduction effect of LC gels was steady, continuous, and would avoid the sharp lowering of IOP to reduce several side effects. These data were supported by the outcomes of the drug release behavior and the cornea penetration studies, indicating LC gels might maintain a sustained-release behavior and strengthen PN absorption to the anterior eye tissues.

### 3.9. Antiglaucoma efficacy

Before establishment of model, the surface of eye was monitored using a slit lamp. As exhibited in Figure 6(A), there was no lesion on corneal surface and even distribution of fundus blood vessels, which indicated that rabbit eyes were healthy and could be used in glaucoma model.

Figure 6(D) displays the curves of miotic effect caused by the pharmacological effect of PN released from LC gels compared with eye drops over 14 days. In the control groups, no significant variations (*p*>.05) are observed in the shrinkage of pupil diameter due to the lack of drug treatments. In contrast, FQ, FH, and eye drops achieve significant reductions (*p* < .05) in contraction effect as 30.56%, 45.45%, and 36.11% at first day, respectively. Furthermore, further decreases of pupil size for LC gels are still noted at the end of the experiments. However, the decreases of pupil diameter for eye drops are observed in six days followed by plateaus in the reduction of pupil size until the end of administration. These observations were coincident with our outcomes of *ex vivo* corneal penetration studies. Obviously, our results indicate the extent of pupil shrinkage in model rabbits in response to pharmacological characteristics, thus reflecting the effect of drug delivery vehicles on the application of therapeutic modality in the management of glaucoma.

Figure 6(E) displays the IOP values in model rabbits, expressed as difference between treatment and normal (i.e. baseline) eyes after 14 days of study. The basic IOP of healthy rabbits were 18.86–20.55 mm Hg before and 28.41–35.32 mm Hg after modeling, which suggested that a successful induction of glaucomatous model. The IOP in control group (glaucomatous rabbits without medication) still maintained a high level and gradually increased to 38 mm Hg after two weeks. These results imply that glaucoma was difficult to self-recover or even may worsen without proper treatment (Zeng et al., 2018). In contrast, the IOP value significantly (*p* < .05) reduced by 57.35%, 51.38%, and 58.10% after a day for model rabbits subjecting to treatment from FQ, FH, and eye drops groups, respectively. For the eye drops groups, the decrease percentages of IOP increased rapidly on the first day and only 11.51% at the end of the treatment, which was due to depletion of the drug as a result of fast release from eye drops. Compared with eye drops, the IOP values of FQ and FH reach to a minimal at around 1st and 2nd day, respectively, where FH remains at low IOP up to six days. The decrease percentages of IOP were still 19.73% and 27.53% for FQ and FH at 14th day, respectively, hinting that the IOP values of LC gels were reduced even more than 2 weeks. These outcomes demonstrated that LC gels could sustainably and steadily reduce IOP in glaucomatous rabbits, may attribute to the prolonged contact time and drug release in the lesion site to prolonged more therapeutical effect. Therefore, LC gels, especially H_2_ gel, had a more remarkable ameliorating effect on IOP-lowering in contrast to commercial products, which was also in line with the results of *in vivo* pharmacokinetic studies.

Interestingly, the animal’s miotic effect and IOP-lowering effect even exceeded 2 weeks in the glaucomatous groups, significantly longer than the normal groups. These results indicate that the removal of the PN in the pathological state was significantly delayed, which may be caused by the imbalance of the production and elimination of aqueous humor (Zeng et al., 2018).

## 4. Conclusions

In this work, biofilm-like PN-loaded LC gels, including Q_2_ phase and H_2_ phase, were prepared for ocular application to treat glaucoma, whose matrix was constituted by PYT/water binary system and PYT/TAG/water ternary system, respectively. Their internal structures were identified by CPLM and SAXS methods to be Pn3m-type Q_2_ and H_2_ mesophase, respectively. Rheological studies revealed that the LC gels showed the properties of pseudoplastic fluid, which was favorable for drug dispersion and absorption on the corneal surface. *In vitro* drug release proved that LC gels showed a sustained release behavior and governed by the Fickian diffusion. The cumulative penetration of drug across the cornea for LC gels was higher than those of traditional eye drops. Furthermore, LC gels possessed a good biocompatibility and prolonged the residence time of the pre-corneal. *In vivo* pharmacokinetic studies attested that compared with PN eye drops, the LC gels could maintain PN concentration in aqueous humor at least 24 hours and significantly enhance the bioavailability of drugs after administration. Finally, *in vivo* pharmacodynamics tests showed that LC gels had an excellent miosis and lowering IOP effect. These outcomes suggest that the LC gels produced in this work might be potentially useful for enhancing cornea transmittance, extending procorneal retention time and improving the efficiency of drug contrasting with eye drops. Thus, it is an effective pharmaceutical strategy to strengthen the ocular absorption of hydrophilic PN for the treatment of glaucoma.
